# A Challenge Called Ogilvie´s Syndrome

**DOI:** 10.7759/cureus.40233

**Published:** 2023-06-10

**Authors:** Luisa Soares Miranda, Carla Silva Gonçalves, Ezequiel Silva, Álvaro Ferreira, João Araújo Correia, Ana Rita Cruz

**Affiliations:** 1 Department of Medical Oncology, Centro Hospitalar Universitário de Santo António, Porto, PRT; 2 Department of Internal Medicine, Centro Hospitalar Universitário de Santo António, Porto, PRT; 3 Department of General Surgery, Centro Hospitalar Universitário de Santo António, Porto, PRT

**Keywords:** abdomen, pain, guidelines, colonic dilatation, ogilvie´s syndrome

## Abstract

Ogilvie´s syndrome is a colonic dilation without any existing mechanical obstruction. The risk factors that cause it are not completely understood, but if untreated, the distension can result in rupture or ischaemic bowel perforation. Additionally, the existing guidelines do not agree with each other about the next steps if conservative treatment fails. We report the case of a 71-year-old woman in whom Ogilvie´s syndrome was particularly difficult to manage, and with it, we try to add clinical data to a field with scarce evidence.

## Introduction

Ogilvie´s syndrome also named acute colonic pseudo-obstruction is a pathologic dilatation of the colon without the presence of an organic obstruction [1,2]. Risk factors leading to Ogilvie´s syndrome are not clearly understood, but it is believed that systemic illness, chirurgic insult and some medications can contribute to a deregulation of the enteric nervous system with autonomic imbalance [3]. Ogilvie´s syndrome is an important source of morbidity and mortality, with ischemia and perforation being the most serious complications. Clinical manifestations of Ogilvie´s syndrome can include abdominal pain and tenderness, nausea, vomiting, and constipation [4].

Evidence concerning the management of Ogilvie´s syndrome is scarce, and even though American and European Guidelines exist, they agree that the first step in the management of Ogilvie’s syndrome should be conservative. American guidelines clearly state that if there are no signs of infection, abdominal tenderness, or free air or cecal diameter > 12 cm, the first-line therapy should include electrolyte correction, avoidance or minimization of narcotics and anticholinergic medication, treatment of a potential infection, bowel rest, alternation position and decompression with nasogastric and/or rectal tubes [4]. Moreover, American guidelines advert that oral osmotic and stimulant laxatives should be avoided because they can worse colon dilation by increasing gas production [4]. European guidelines do state that a prompt endoscopic compression should be done if the cecal diameter is higher than 12 cm and if Ogilvie’s syndrome is present for more than 4-6 days [5,6]. European guidelines recommend endoscopic decompression of the colon when conservative treatment fails but highlight that no study proved the superiority of endoscopic decompression in relation to neostigmine [5,6]. Additionally, European guidelines state that endoscopic decompression after recurrence may still be effective, but state that literature is not clear concerning it and more evidence is needed [5,6]. Conversely, American guidelines recommend that after conservative treatment failure, neostigmine should be used, and that endoscopic colonic decompression should be considered only in patients in whom neostigmine therapy is contraindicated or ineffective [4]. The low number of studies concerning the management of Ogilvie's makes it difficult to build recommendations based on high levels of evidence. With this in mind, we decided to publish our experience managing a particularly challenging case of Ogilvie's syndrome.

## Case presentation

**Figure 1:**
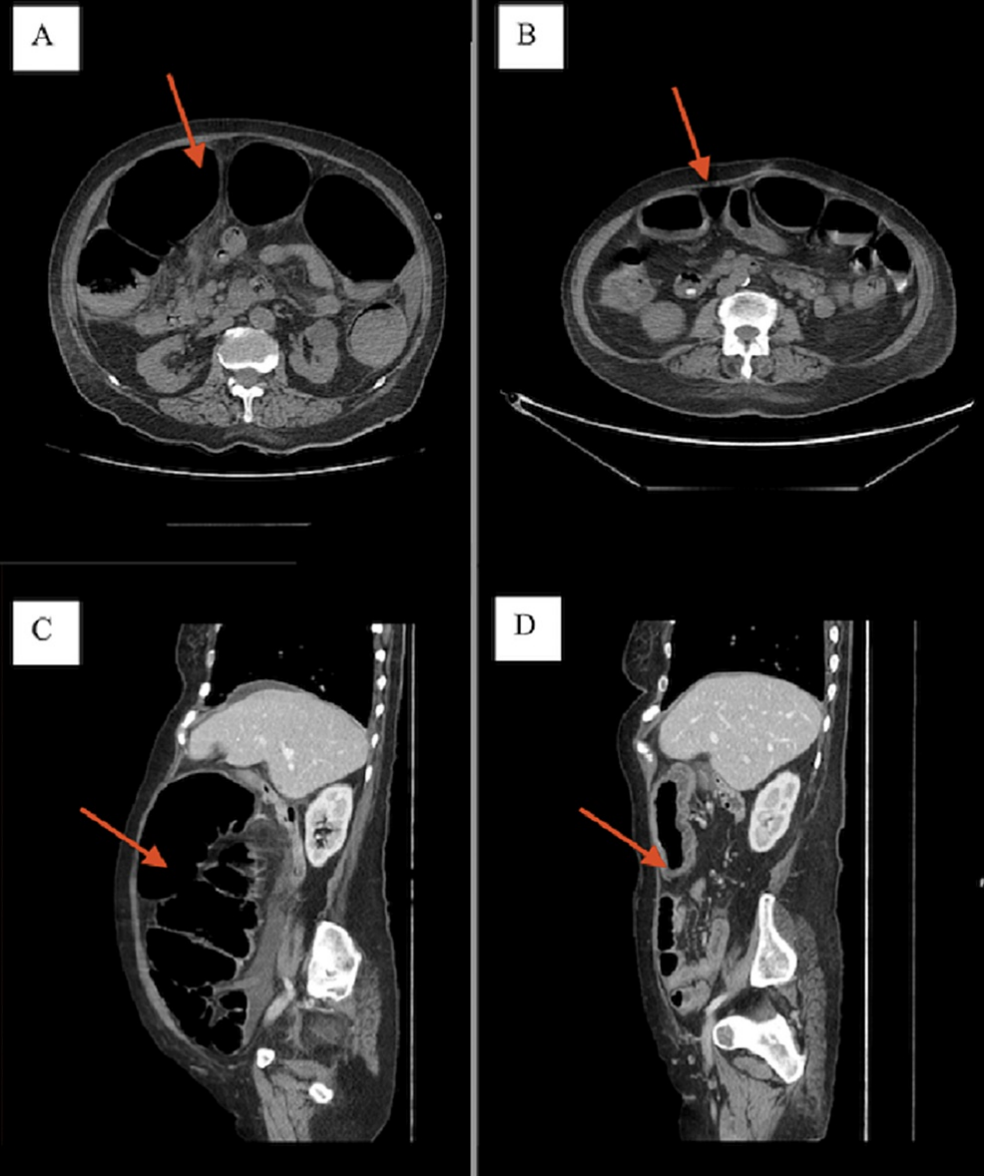
Abdominal scan done at patients’ admission (A and C) and patients’ discharge (B and D) Images A and C show marked distention of the colon. In images B and D, we can observe an improvement in colonic distention.

A 71-year-old woman was observed in the emergency room due to abdominal distention and peri-umbilical pain of high intensity (8 in 10 using the numeric pain rating scale). Her medical history was significant for psychiatric disease (with strong maniac and psychotic symptoms in study by psychiatry), hypertension, and dyslipidemia. Medications included lisinopril, simvastatin, paliperidone, valproate, trazodone, lorazepam, tapentadol, betahistine, tramadol, and paracetamol. On examination, a significantly distended and painful abdomen was observed. Abdomen X-ray revealed a large abdominal distention. A computerized tomography scan was done (Figure 1) to exclude mechanical obstruction that showed a distention (maximum 84 mm) present in the entire colon extending till the caecum, without a transition point (Figures 1A, 1C).

On laboratory investigations, hypokalemia and hyponatremia were observed, with no other lab abnormalities, explained by the profuse defecation observed in the emergency setting and some degree of dehydration. During the first 72 hours of hospitalization, conservative treatment was initiated, with fasting, nasogastric drainage, and electrolyte correction. Seventy-two hours after conservative treatment patient’s abdomen was still very distended and hyper tympanic, despite the pain resolution. On examination, there was a significantly distended abdomen and the abdominal X-ray persisted with large abdominal distention. The hemogram and biochemical parameters were under normal values. The patient was allowed, subsequently, to begin a non-fiber diet and the nasogastric drainage was withdrawn. Discontinuation of psychiatric medications was discussed, but not pursued due to the patient’s strong psychotic symptoms with difficult control over the years. A decompressive colonoscopy was conducted, due to the failure of a conservative approach, with a transitory resolution of the colonic distention that lasted less than 24 hours. A second decompressive colonoscopy was performed with similar results. Distension exacerbation and complaints of abdominal pain together with radiologic worsening, of colonic distention, made us opt for pharmacologic treatment with neostigmine, with some response. In 24 hours, abdominal distension worsened again. Due to failure of endoscopic and pharmacologic treatment, after a first approach of 72 hours of conservative treatment, it was decided to step back to conservative attitudes with fasting, incentive to walk, and alternation of position upon lying down. Decompression with nasogastric and rectal tube and enemas (two times per/day) with clinical and imagological improvement on the fifth day (Figures 1B, 1D). A gradual introduction of oral diet was done, first only with liquids, and sequential with a non-fiber diet, and no recurrence was observed.

## Discussion

The estimated risk of spontaneous perforation, in Ogilvie´s syndrome, is 3% with an associated mortality rate of 50 percent [2]. Management of Ogilvie´s syndrome can be challenging, additionally, the fact that evidence for its management is scarce and the existing guidelines do not agree with each other in what to do if conservative treatment fails can add more difficulty to its management [4-6]. European Society of Gastrointestinal Endoscopy recommends considering endoscopic colon decompression in patients with Ogilvie’s syndrome that are not improving with conservative treatment [6]. On the other hand, the American Society of Colon and Rectal Surgeons suggests that pharmacologic treatment with neostigmine is indicated when Ogilvie´s syndrome does not solve with conservative therapy and that endoscopic colonic decompression should be pondered in patients in whom neostigmine therapy is contraindicated or unsuccessful [4]. As no prospective direct comparison between endoscopic decompression and neostigmine treatment exists, the superiority of one of these options in comparison to the other, in patients with Ogilvie’s syndrome that are not improving with conservative treatment, cannot be assumed [6].

The etiology of Ogilvie´s syndrome is not entirely understood but appears to be multifactorial [7]. It is thought that some medications might contribute to it [7]. Our patient has been under substantial psychiatric medication (paliperidone, valproate, trazodone, lorazepam), a risk factor for Ogilvie´s syndrome, that could not be stopped or changed. Consecutive recurrence of the colonic distention after conservative, endoscopic, and pharmacologic approaches was not expected and was difficult to manage. The re-introduction of conservative treatment was, in this case, important. One previous retrospective study suggested that interventional treatments do not appear to be more effective than conservative treatment [1] and recommend that clinicians should be encouraged to apply elementary actions of supportive care while handling Ogilvie's syndrome, such as correcting electrolyte abnormalities, weaning narcotics, providing a nasogastric or rectal tube for decompression, and encouraging ambulation when possible [1]. Some recommend that, once the diagnosis of Ogilvie´s syndrome is made, it is important to determine the treatment plan; urgency in treatment includes a diameter of more than 12 cm, which is often considered less responsive to conservative therapy [8]. Our case report builds upon and expands these previous results by suggesting that even after endoscopic and pharmacologic approaches failure, it might be worth implementing the conservative approach again. Consequently, this case makes us think if aggressive conservative treatment should be kept longer before thinking of other therapeutic options such as endoscopic or pharmacologic treatment.

## Conclusions

Scientific evidence for the management of Ogilvie´s syndrome is scarce, and the existing guidelines do not agree with each other regarding what to do if conservative treatment fails. Our patients had strong psychiatric medication (paliperidone, valproate, trazodone, lorazepam), a known risk factor for Ogilvie´s syndrome. That medication could not be stopped or changed. The successive reappearances of the colonic distention after different approaches were not expected and were challenging to manage. This case makes us think if aggressive conservative treatment should be kept longer before considering other therapeutic options as endoscopic or pharmacologic treatment if no signs of complicated disease are present.
